# Sodium butyrate promotes apoptosis in breast cancer cells through reactive oxygen species (ROS) formation and mitochondrial impairment

**DOI:** 10.1186/s12944-017-0593-4

**Published:** 2017-11-02

**Authors:** Vahid Salimi, Zahra Shahsavari, Banafsheh Safizadeh, Ameinh Hosseini, Narges Khademian, Masoumeh Tavakoli-Yaraki

**Affiliations:** 1 Department of Virology, School of Public Health, Tehran University of Medical Sciences, Tehran, Iran; 2 Department of Laboratory Medicine, Faculty of Paramedical Sciences, Shaheed Beheshti University of Medical Sciences, Tehran, Iran; 3 Department of Biochemistry, Faculty of Medicine, Birjand University of Medical Sciences, Birjand, Iran; 4 Department of Biochemistry, School of Medicine, Iran University of Medical Sciences, Tehran, Iran

**Keywords:** Sodium butyrate, Apoptosis, Cell cycle, Reactive oxygen species, Caspase, Mitochondrial membrane potential (Δψm)

## Abstract

**Background:**

Sodium butyrate (NaBu) is a short-chain fatty acid which serves as a histon deacetylase inhibitor and has received considerable interest as a possible regulator of cancer cell death. The regulatory effect of NaBu on cancer cell growth or death has yet to be illustrated in many cancers including breast cancer. This study is aimed to elucidate the possible effect of NaBu on regulation of breast cancer growth and apoptosis.

**Methods:**

The cytotoxic effect of NaBu on the growth of breast cancer cells (MCF-7 and MDA-MB-468) and normal breast cells (MCF-10A) was determined using MTT assay. Annexin-V-FITC staining and PI staining were performed to detect apoptosis and cell cycle distribution using Flow cytometry, the level of mitochondrial membrane potential (Δψm), Reactive oxygen species (ROS)formation and caspase activity were determined accordingly.

**Results:**

Based on our data, NaBu induced a dose and time-dependent cell toxicity in breast cancer cells which was related to the cell cycle arrest and induction of apoptosis. The impact of NaBu on MCF-10A cell toxicity, cell cycle distribution and apoptosis was inconsiderable. NaBu-elicited apoptosis was accompanied by the elevated level of ROS, increased caspase activity and reduced mitochondrial membrane potential (Δψm) in MCF-7 and MDA-MB-468 cells and with no effect on the above mentioned factors in MCF-10A cells.

**Conclusions:**

Our study provided insight in to the role of NaBu on the regulation of breast cancer cell growth and lighten up the pro-apoptotic activity of NaBu.

## Background

The balance between apoptosis and proliferation determines the homeostasis of cell growth. Cancer cell evades apoptosis to accelerate its proliferation and progression [1]. Accordingly, the molecular mechanisms responsible for the loss of apoptosis and gain of proliferation is critical for controlling cancer cell growth [2]. Amongst the epigenetic regulation mechanisms, the acetylation status of genes which is regulated by Histone acetyltransferases (HAT) and Histone deacetylases (HDAC) is served as a critical regulatory mechanism for controlling gene expression and chromatin structure [3]. Accordingly, Development of histone deacetylase inhibitors (HDACi) as promising anticancer targets has received considerable interests recently [4, 5]. Additionally, attentions are expanding on the promising effect of lipids on the cell proliferation and death [6–8]. Sodium butyrate (NaBu), one of the well-studied HDACi, is a short-chain fatty acid and the byproduct of carbohydrate metabolism in the gut [9]. It emerges as an inhibitor of HDAC and involves in various cellular process such as cellular proliferation, differentiation and gene expression [10]. Several mechanisms are proposed to be involved in the regulation of cancer cell growth induced by Sodium butyrate including the inhibition of DNA double strand break repair and stress oxidative [9, 11]. It has been shown that sodium butyrate suppress oncogene Bim1 in tongue cancer [12]. Moreover, sodium butyrate induced both intrinsic and extrinsic pathway of apoptosis in human pancreatic cancer cell lines [13]. However the relevance of Sodium butyrate and cancer cell growth has yet to be investigated in many cancers. The heavy burden of breast cancer-related mortality and morbidity [14] on one hand and lack of sufficient evidences about the effect of sodium butyrate on breast cancer cell growth on other hand, provoked us to unravel the mechanism by which sodium butyrate affects the growth of tightly cohesive MCF-7 and triple negative highly metastatic MDA-MB-468 breast cancer cells and/also MCF-10A as the normal breast cells. To aim this, the dose and time dependency of breast cancer cell toxicity induced by sodium butyrate was studied. Also, the effect of sodium butyrate on the cell cycle distribution, intracellular formation of Reactive oxygen species (ROS), the caspase 3 and 8 activity, mitochondrial membrane potential estimation and induction of apoptosis was further assessed.

## Methods

### Chemical reagents and materials

RPMI 1640, trypsin/EDTA, Nacl/Pi, DMEM-F12, penicillin and streptomycin were purchased from Gibco (Rockville, USA). The annexin-V-FITC apoptosis detection kit, propidium iodide (PI), MTT [3-(4,5-dimethyltiazol-2-yl)-2,5-diphenyltetrazolium bromide], JC- 1, dimethylsulfoxide, hydrocortisone, EGF, Insulin and Sodium butyrate were obtained from Sigma-Aldrich (Munich,Germany). Caspase-3 and caspase-8 colorimetric assay kits were obtained from BioVision (BioVision, Inc. Milpitas, CA USA). Fluorescent Reactive Oxygen Species detection kit was obtained from Marker Gene Technologies (MGT, Inc., USA).

### Cell culture

The human breast cancer cell lines, MCF-7 and MB-MDA-468, were obtained from Pasture Institute of Iran. Cells were cultured in RPMI 1640 medium containing 10% (*v*/v) fetal bovine serum, 100 U/ml of penicillin and 100 μg/ml of streptomycin. Cells were maintained at 37 °C with an atmosphere of 5% CO_2_ and 100% humidity. To passage the cells, cells were exposed to trypsin which facilitate cell separation at the confluence of 70–100%. The collected cells were used freshly or were frozen and stored at −80 °C for further experiments. The MCF10A breast normal cells were purchased from Pasture Institute of Iran. Cells were cultured in Dulbecco’s modified Eagle’s medium and F12 medium (DMEM-F12) which was supplemented with horse serum (5%), hydrocortisone (0.5 μg/ml), EGF (20 ng/ml) and insulin (10 μg/ml) and 100 U/ml of penicillin and 100 μg/ml of streptomycin. The maintained condition was provided at 37 °C with an atmosphere of 5% CO_2_ and 100% humidity [15]. The 3.0 mL 0.05% trypsin with 0.53 mM EDTA was used for cell passage.

### Cell viability assay

The cytotoxicity of sodium butyrate on breast cancer cells was studied using MTT assay.

Briefly, MCF-7 and MDA-MB-468 were seeded in 96-well plates at 5*10^3^ cell/well in RPMI 1640 (supplemented with 10% fetal bovine serum, 100 U/ml of penicillin and 100 μg/ml of streptomycin) also MCF-10A cells were cultured in DMEM-F12 (supplemented with 5% horse serum, hydrocortisone (0.5 μg/ml), EGF (20 ng/ml), insulin (10 μg/ml) and 100 U/ml of penicillin and 100 μg/ml of streptomycin) in 5% CO2 at 37 °C and after being nearly confluent they were treated with different concentrations (0.1–20 mM) of sodium butyrate and incubated for 24, 48 and 72 h. Based on MTT assay protocol, 20 μl of MTT (5 mg/ml in PBS) was added to each well and incubated for 4 h at 37 °C, the supernatant of each well was removed and 200 μl of dimethylsulfoxide (DMSO) was added to each well to dissolve formazan crystals. The absorbance values were read using a microplate reader (Bio-Rad, Hercules,CA, USA) at 570 nm, the percentage of cell viability was reported according to comparison of treated groups and vehicle control groups (DMSO). The experiment was repeated several times and data are representative of at least three distinct experiments.

### Assessment of apoptosis

In order to verify whether sodium butyrate can induce apoptosis, Annexin-V-FITC kit was used and further detected by flow cytometry according to the manufacturer’s instructions. Cells were seeded in 6 well plates and harvested at the density of 1*10^6^ cell/ml. Following washing by PBS, cell pallets were suspended in 500 μl of 1 × binding buffer then each sample were exposed to 5 μl of annexin-V-FITC and, 5 μl of PI and incubated for 10 min at room temperature subsequently analyzed by FACSCalibur flowcytometer (BectonDickinson, SanJose,USA) and its supplied software (BD CellQuest software).

### Cell cycle analysis

One of the accurate methods for analyzing DNA content and cell cycle distribution, is PI staining. Briefly, cells were treated with different concentrations of sodium butyrate for 48 h in RPMI 1640 (serum free) for MCF-7 and MDA-MB-468 and in DMEM-F12 (serum-free) for MCF-10A then fixed with 70% (*v*/v) ice-cold ethanol upon harvesting and washed twice with ice-cold PBS and stained with 0.1 mg/ml of RNase A (Sigma) and 0.05 mg/ml of PI (sigma) for 1 h at 37 °C in the dark. Cells were assessed by ACSCalibur flow cytometer (BectonDickinson, SanJose,USA) and analyzed by its supplied software (BD CellQuest software). The percentages of cell population in various cell cycle phases were reported respectively. Apoptotic cells are accumulated in sub-G1 phase.

### Measurement of caspase-3 activity

The activity of caspase 3 following treatment with sodium butyrate was determined using Caspase-3 colorimetric Assay Kit (BioVision Inc. Milpitas, CA USA).). Cells were treated with 5 and 10 mM of sodium butyrate for 48 h and cells were lysed in chilled lysis buffer and incubated for 10 min following gentle centrifugation 50 μM of reaction buffer containing 10 mm DTT and 5 μM of DEVD-pNA substrate (200 μM) was added to each sample and incubate for 120 min at 37 C. the activity of caspase 3 was quantified by measuring the emission at 405 nm.

### Measurement of Caspase-8 activity

The activity of caspase 8 in live cells were assessed using Caspase-8 Detection Kit. According to the provided instruction, cells were cultured in 24-well plate and pretreated for 3 h with 20 μM Z-VAD-FMK or 50 μM Nec-1, then cells exposed to 5 and 10 mM of sodium butyrate for 48 h. 300 μl of each treated and untreated cells were taken and aliquot into separate tubes. 1 μl of FITC-IETD-FMK, added to each tube and incubate for 0.5–1 h in an incubator with 5% CO2 at 37c. Notably, FITC-IETD-FMK is a labeled inhibitor of activated caspase-8 in live cells which is non-toxic and cell permeable. Following two steps of centrifugation, cell pallets were suspended in wash buffer and subjected to black microtiter plate and their fluorescence intensity were measured at excitation 485 nm and emission 535 nm.

### Measurement of intracellular reactive oxygen species (ROS)

The level of intracellular ROS formation including hydrogen peroxide (H2O2), hydroxyl radical (OH•), and hydroperoxides (ROOH) were measured using 2′, 7′-dichlorofluorescin diacetate (DCFH-DA) as a fluorescent probe. Based on the method, cells were cultured in 96 cell well plate, and DCFH-DA (20 μM working substrate solution) was added in culture medium and cells were incubated at 37 °C for 45 min and treated with 1, 5 1nd 10 mM of sodium butyrate following removal of substrate solution. The level of ROS formation was determined according to the level of generated Fluorescence at an excitation and emission wavelength of 485 nm and 528 nm using a fluorescent micro plate reader (BioTek Synergy Ht, Winooski, Vermont, USA) and the respective ROS level was expressed as RFU.

### Measurement of mitochondrial membrane potential (Δψm)

In order to determine the involvement of mitochondrial in sodium butyrate- induced apoptosis, JC-1 staining was applied as a method of choice. Potential dependent accumulation of JC-1 as a lipophilic cationic dye is characterized by fluorescence emission shift from green (~520 nm) to red (~590 nm). Accordingly, a decrease in the red/green fluorescence intensity ratio indicates mitochondrial depolarization. To this aim, cells were cultured in 96-well plate, and treated with 1, 5 and 10 mM of sodium butyrate for 48 h. Following removal of media, 10 μl of staining solution was added to each well and incubated for 15 min at 37 °C, 5% CO2. Plate was subjected to several centrifugation and replacement of media by assay buffer, then assessed on a fluorescent micro plate reader (BioTek Synergy Ht, Winooski, Vermont, USA). The filters were set as: 485 nm excitation/ 528 nm emission (green) and 530 nm excitation/ 590 nm emission (red).Data were presented as the ratio of fluorescent intensity of green (590 nm, emission of J-aggregate form) to fluorescent intensity of red (530 nm, emission of JC-1 monomeric form).

### Statistical analysis

For analyzing and comparing data, Non-parametric one-way analysis of variance (ANOVA) with Dennet’s post hoc test and Tukey’s post hoc test were applied using software GraphPad Prism. To determine specificity and accuracy, all experiments were performed in triplicate and repeated at least three times. Data are presented as mean ± SD and differences were taken significant for *P* < 0.05, *P* < 0.01 and *P* < 0.001. The statistical differences were determined by asterisk and shown as *P < 0.05, **P < 0.01, ***P < 0.001 in corresponding figures.

## Results

### Sodium butyrate inhibited breast cancer cell growth in a time and dose dependent manner

**Fig. 1:**
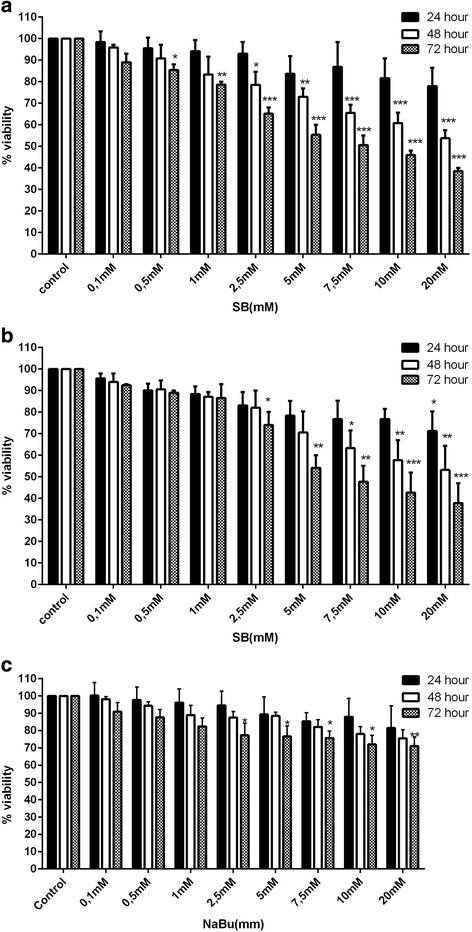
Sodium butyrate inhibited breast cancer cell proliferation. MCF-7(**a**), MDA-MB-468(**b**) and MCF-10A (**c**) cells were treated with different concentrations of sodium butyrate (0.1–20 mM) for 24,48, 72 h. The Percentages of viable cells were determined using MTT assay. Inhibition of cell growth was occurred in time and dose-dependent manner.Data represent Mean ± SD of three separate experiments. The Statistical differences between treated and untreated groups were analyzed by ANOVA (* = *P* < 0.05, ** = *P* < 0.01, *** = *P* < 0.001)

To explore the effect of sodium butyrate on breast cancer and normal cell growth, cells were exposed to the different concentrations of sodium butyrate (0.1–20 mM) for 24, 48, 72 h and the viability of cells were assessed using MTT assay. Our results have shown the significant decrease in the percentage of viable breast cancer cells (MCF-7 and MDA-MB-468) treated with increasing concentrations of sodium butyrate. The pattern of sodium butyrate-induced cell growth inhibition was in a dose and time dependent manner for both MCF-7 (Fig. 1a) and MDA-MB-468 (Fig. 1b). Treatment of MCF-7 cells by 5 mM and 10 mM of sodium butyrate for 48 h resulted in reduction of cell viability about 27% and 40%, respectively (Fig. 1a). The cytotoxic effect of 5 and 10 mM of sodium butyrate on MDA-MB-468 cells was observed as the amount of 30% and 43% reduction in cell viability after 48 h, respectively (Fig. 1b). To provide more accurate comparison, MCF-10A cells were treated with the same concentrations of sodium butyrate (0.1–20 mM) for 24, 48, 72 h and the subsequent cytotoxic effect was significantly lower comparing to the breast cancer cells. As it is shown in Fig. 1c, sodium butyrate induced cell death at its higher concentrations after 72 h in MCF-10A which the observed rate of dead cells can be due to the longtime exposure of cells to the sodium butyrate.

### Sodium butyrate induced apoptosis in breast cancer cells

**Fig. 2:**
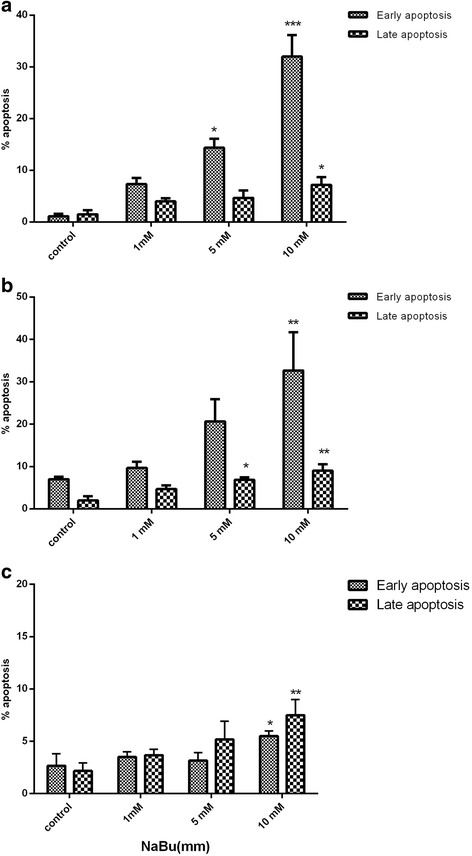
Sodium butyrate induced apoptosis in breast cancer cells. Cells were treated with 1, 5 and 10 mM of sodium butyrate for 48 h and apoptosis was detected by annexin-V and PI staining and analyzed by flow cytometry for MCF-7 (**a**), MDA-MB-468 (**b**) and MCF-10A (**c**). A significant increase in the percentages of early apoptotic cells were detected in both cells. Data represent Mean ± SD of three separate experiments. . Statistical differences between different groups were analyzed by ANOVA (* = *P* < 0.05, ** = *P* < 0.01, *** = *P* < 0.001)

Annexin-V and PI double staining method was applied as a method of choice to determine whether the cytotoxic effect which was observed from sodium butyrate was related to the induction of apoptosis. To aim this, cells were treated with 1, 5 and 10 mM of sodium butyrate for 48 h, and analyzed by Flowcytometry based on the recommended method. According to the protocol, early apoptotic cells are annexin-V positive, PI negative cell and late apoptotic cells were recognized as annexin-V positive, PI positive. Our results have revealed a significant increase in the percentage of early and late apoptotic cells following treatment with increasing concentrations of Sodium butyrate for both MCF-7 (Fig. 2a) (*P* < 0.001) and MDA-MB-468 (Fig. 2B) (*P* < 0.001) however no significant elevation was observed in the percentage of apoptotic cells in MCF-10A cells. Based on our data the percentage of early apoptotic cells after treatment by 5 mM of sodium butyrate was 14.66% for MCF-7 (Fig. 2a) and 22% for MDA-MB-468 (Fig. 2b), and 3% for MCF-10A normal cells(Fig. 2c), respectively.

### Cell cycle arrest was induced following treatment by sodium butyrate

**Fig. 3:**
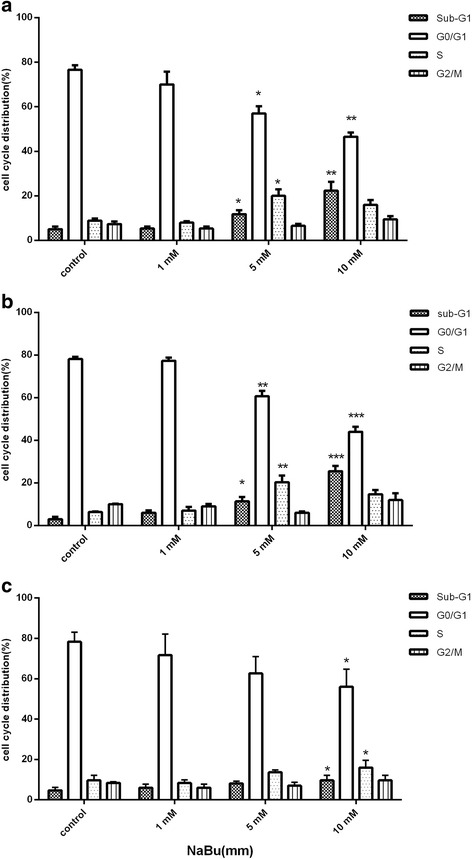
Sodium butyrate induced cell cycle arrest in breast cancer cells. Cells were treated with sodium butyrate (1, 5, 10 mM) for 48 h and cell cycle distribution were analyzed by flow cytometry using PI staining for MCF-7 (**a**), MDA-MB-468 (**b**) and MCF-10A (**c**).The accumulation of cells in sub-G1 phase was increased significantly in both cell lines. Data represent Mean ± SD of three separate experiments. Statistical differences between different groups were analyzed by ANOVA (* = *P* < 0.05, ** = *P* < 0.01, *** = *P* < 0.001)

One of the hallmarks of apoptosis is changes in the pattern of cell cycle distribution which is observed as an accumulation of cells in the Sub-G1 phase. To evaluate the impact of sodium butyrate on the cell cycle progression, cells were exposed to different concentrations of sodium butyrate (1, 5 and 10 mM) for 48 h and analyzed by Flowcytometry according to the mentioned protocol. Our analysis has shown the significant elevation in the percentage of accumulated cells in the sub-G1 phase which was observed in MCF-7 and MDA-MB-468 cells however the effect of sodium butyrate on MCF-10A cell cycle distribution was inconsiderable (Fig. 3). Based on our results, the rate of cells in sub-G1 phase were significantly enhanced in a dose-dependent manner for both MCF-7 (Fig. 3a) (*P* < 0.001) and MDA-MB-468 (Fig. 3b) (*P* < 0.001) cells. The percentage of cells at Sub-G1 phase was slightly increased after 72 h in MCF-10A cells which shows less effectiveness of sodium butyrate on these cells (Fig. 3c).

### The activity of caspase 3 and 8 increased toward sodium butyrate-elicited apoptosis in breast cancer cells

**Fig. 4:**
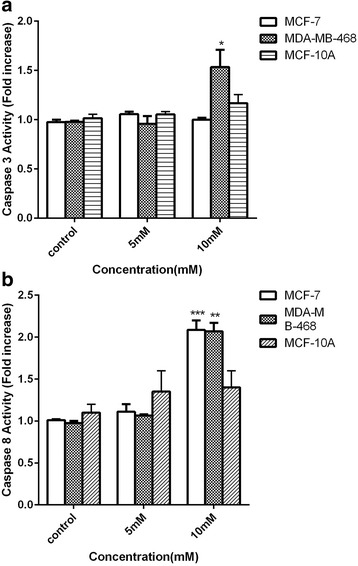
Sodium butyrate increased caspase activity in breast cancer cells. Cells were treated with sodium butyrate (5 and 10 mM) for 48 h and the activity of caspase 3 (**a**) and caspase 8 (**b**) was measured accordingly. The activity of caspase-8 was elevated in both cell lines. However, the caspase 3 activity was increased in MDA- MB-468 and remained unchanged in MCF-7 cells and MCF-10A cells. Data represent Mean ± SD of three separate experiments. Statistical differences between different groups were analyzed by ANOVA (* = *P* < 0.05, ** = *P* < 0.01, *** = *P* < 0.001)

Given the remarkable role of caspase cascade as an executors of apoptosis, the possible involvement of caspase 3 and caspase 8 in sodium butyrate-induced apoptosis in breast cancer and normal cells were evaluated. To address this, cells were treated with 5 and 10 mM of sodium butyrate for 48 h and subjected to further analysis for contribution of caspase 3 and 8. As it is indicated in Fig. 4, a significant increase in the activity of caspase 8 was observed at 10 mM concentration of sodium butyrate for MDA-MB-468 (*P* < 0.01) and MCF-7 (*P* < 0.001) (Fig. 4b) however the level of caspase 8 activity remained unchanged at the mentioned time and dose in the MCF-10A cells (Fig. 4b). Treatment of cells by sodium butyrate resulted in the elevation of caspase 3 activity in MDA-MB-468 cells (Fig. 4a) however no significant elevation was observed in the activity of caspase 3 in MCF-7 which is in line with other studies and confirmed the fact that induction of apoptosis in MCF-7 cells is occurred in a caspase 3 independent manner. In accordance, no significant elevation in the level of caspase 3 activity was observed in MCF-10A cells.

### ROS formation was elevated following sodium butyrate-induced apoptosis

**Fig. 5:**
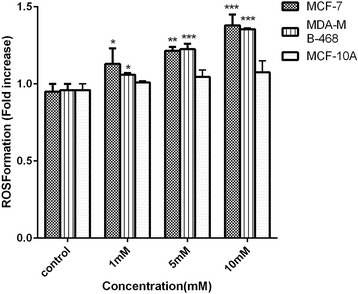
Sodium butyrate stimulates ROS formation in breast cancer cells. MCF-7, MDA-MB-468 and MCF-10A cells were treated with sodium butyrate (1, 5, 10 mM) for 48 h and subjected to ROS measurement as described in material and methods. A significant dose-dependent increase in the level of intracellular ROS was observed in both MCF-7 and MDA-MB-468 which remained unchanged in MCF-10A. Data represent Mean ± SD of three separate experiments. Statistical differences between different groups were analyzed by ANOVA (* = *P* < 0.05, ** = *P* < 0.01, *** = *P* < 0.001)

It is well evidenced that intracellular ROS level is increased in apoptotic cells which sustain stressful conditions. To investigate the possible generation of ROS in response to sodium butyrate, cells were treated with different concentrations of sodium butyrate (1, 5 and 10 mM) for 48 h and the level of ROS production was assessed accordingly. Based on the results, a significant increase in the level of intracellular ROS was observed in both MCF-7 (*P* < 0.001) and MDA-MB-468 (*P* < 0.001) which was occurred in a dose-dependent manner (Fig. 5) however the level of ROS production was remained approximately unchanged in MCF-10A cells..

### Mitochondria is involved in the sodium butyrate-induced apoptosis in breast cancer

**Fig. 6:**
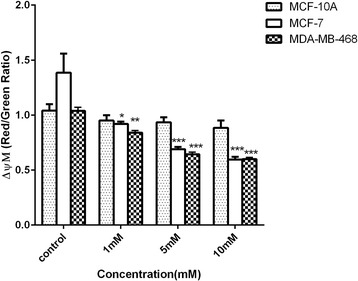
Sodium butyrate stimulates mitochondrial membrane potential (ΔΨm) depletion in breast cancer cells. MCF-7, MDA-MB-468 and MCF-10A cells were treated with sodium butyrate (1, 5, 10 mM) for 48 h and the level of mitochondrial membrane potential (ΔΨm) was determined using JC-1 protocol. Sodium butyrate induced a significant decrease in mitochondrial membrane potential (ΔΨm) in a dose dependent manner. Data represent Mean ± SD of three separate experiments. Statistical differences between different groups were analyzed by ANOVA (* = *P* < 0.05, ** = *P* < 0.01, *** = *P* < 0.001)

Mitochondria membrane potential (ΔΨm) reflects mitochontrial function and decrease in the level of (ΔΨm) is considered as one of the markers of apoptosis. In order to characterize the possible contribution of mitochondria in sodium butyrate-induced apoptosis, cells were treated with 1, 5 and 10 mM of sodium butyrate for 48 h and incubated with JC-1 as a lipophilic labeled dye which is able to enter mitochondria and shift color from green to red while membrane potential is increased. JC-1 aggregates in healthy cells with intense red color and tends to stay as monomer and show green color in apoptotic cells in which the membrane potential is low. The red to green shift reflects loss in mitochondrial membrane potential. Based on our results, as indicated in Fig. 6, sodium butyrate significantly induced mitochondrial membrane potential (ΔΨm) depletion in a dose dependent manner in MCF-7 (*P* < 0.001) and MDA-MB-468 (*P* < 0.001) cells and no significant changes in MCF-10A normal cells.

## Discussion

The homeostasis of cancer development and progression is highly depend on the balance of cancer cell growth and death [16, 17]. Apoptosis is a kind of programmed cell death which contributes to the physiological and normal cell life [1]. Abnormalities in the regulation of cell death as well as cell growth leads to the outburst of complex diseases such as cancer [18, 19]. Over-proliferation of cells beside attenuation of apoptosis and cell removal are considered as obvious characters of cancers [2]. Accordingly, induction of apoptosis is proposed as an efficient molecular approaches to remedy cell death balance when apoptosis is diminished [1]. Sodium butyrate is a short-chain fatty acid and by product of carbohydrate metabolism in the gut and consider as a potent HDACi which emerges as an anticancer agent for some cancers [13]. Despite of many attempts to determine the possible anticancer effect of sodium butyrate, its exact effect has yet to be elucidated in many cancers which their data will shed more light on the relevance of sodium butyrate and its biological roles. To address this, we set up a connected set of experiments to characterize the possible anticancer effect of sodium butyrate on breast cancer cell growth as well as normal breast cells. The results of the current study also our previous study [20] have revealed that sodium butyrate deceased the rate of viable breast cancer cells in a dose and time dependent manner. In our previous study the involvement of 15-lipoxygenase in the regulation of breast cancer cell growth induced by sodium butyrate was explored [20], however in the present study the exact mechanism underlying sodium butyrate-induced cell death in human breast cancer is perused. In support of this, the appropriate range of sodium butyrate concentrations (0.1–20 mM) at different time intervals were investigated. Accordingly, 5 m M of sodium butyrate reduced the percentage of viable MCF-7, MDA-MB-468 and MCF-10A cells about 27%, 30% and 10%, respectively which is in line with the effect of sodium butyrate on other cancer cells [9]. Our results showed that the cytotoxic effect of sodium butyrate was related to the induction of apoptosis in both cell lines however the rate of apoptosis induction via sodium butyrate in MCF-10A was significantly lower comparing to the breast cancer cells. Natoni et all, have shown that sodium butyrate can also sensitize human pancreatic cancer cell lines to both intrinsic and extrinsic pathway of apoptosis [13]. To determine whether cell cycle distribution is affected following sodium butyrate treatment, we used flowcytometry with the protocol of PI staining in this regards. Our results have shown the significant accumulation of both MCF-7 and MDA-MB-468 in sub-G1 phase which accounts as a hallmarks of apoptosis although this accumulation was not considerably observed in MCF-10A cells. Caspases are endoproteases responsible for cysteine-dependent hydrolyzing of their targets. Their function results in generating active signaling molecules that are involved in processing apoptosis and inflammation [2]. Different types of caspases contribute to the apoptotic pathway amongst caspase 8 is classified as an initiator and caspase 3 as an executor caspase [1]. We demonstrated that the activity of caspase 8 was increased in a dose-dependent manner in both breast cancer cell lines however the level of caspase 8 activity was remained unchanged in normal breast cells. Notably, the activity of caspase 3 was just increased in MDA-MB-468 but remained unchanged in MCF-7 and MCF-10A in response to sodium butyrate. The caspase 3-independent manner of MCF-7 for induction of apoptosis has been suggested previously [21] and our data further confirmed this hypothesis. In order to characterize the mechanism underlying sodium butyrate-elicited apoptosis, we have shown that sodium butyrate can increase the level of intracellular ROS production in both MCF-7 and MDA-MB-468 cell lines but not changed in MCF-10A. It has been shown that ROS accumulation results in impairment of some cellular functions and leads to the promotion of apoptosis [22]. Our data is consistent with the data presented in the study of Louis et all that sodium butyrate induced apoptosis via oxidative stress [11]. Interestingly, energy metabolism of breast cancer cells can be affected by sodium butyrate however sodium butyrate manipulates metabolic pathways differently through attenuation of glycolysis and glucose 6-phosphate dehydrogenase activity in MCF-7 and activation of glucose 6-phosphate dehydrogenase and consumption of oxygen in MDA-MB-231 [23]. It was postulated that mitochondria might be one of the targets of ROS attacks which its membrane potential will be influenced [24]. In accordance, we measured the mitochondrial membrane potential (Δψm) following treatment by sodium butyrate and our data has shown a dose-dependent depletion of mitochondrial membrane potential in both cell lines however remained unchanged in MCF-10A cells which provide insight in to the involvement of ROS and mitochondrial membrane impairment in sodium butyrate-exhibited apoptosis. It has been shown that HDAC inhibition induced apoptosis via upregulation of TRPM2 (Transient receptor potential cation channel, subfamily M, member 2) expression in bladder cancer cells [25]. Also it has been shown that treatment by sodium butyrate induced chromatin relaxation and suppressed DNA double strand break both in MCF-7 and non-cancerous human embryonic kidney293 (HEK293) cells which sensitize cancer cells to death [9]. It has been shown that p21 is not the target of HDACi for processing apoptosis [26]. It is also shown that sodium butyrate exerts its anti tumorgenic effect via pharmalogical silencing of oncogene Bim1 in tongue cancer [12]. Also sodium butyrate mediates colon cancer cell differentiation through galectins-1 and -3 and induced expression and function of alkaline phosphatase [27]. It can also sensitize human pancreatic cancer cell lines to both intrinsic and extrinsic pathway of apoptosis [13]. According to our results, the minimum effective dose of sodium butyrate which induced breast cancer cell toxicity was not physiologically relevant. We have set connected series of experiments with different doses of sodium butyrate however the concentration of 5 mM was the confident dose. The combination of lower doses of sodium butyrate with a pro-apoptotic agents might help to favor the benefits of sodium butyrate in induction of apoptosis. We have shown that 15-lipoxygenase product has synergistic effect with sodium butyrate in induction of toxicity in breast cancer cells [20]. Our results were in line with previous studies about the effectiveness of sodium butyrate on breast cancer cell regulation [9, 28–30], however our study aimed to investigate complete panel of elements which might be involved in the induction of apoptosis insisting on the role of mitochondria and ROS which were not considered in the previous studies. Sodium butyrate belongs to the family of short chain fatty acids which contains members such as propionate which considering their effect on cancer cell growth and mechanism of cell growth regulation would provide remarkable data and is worth for further consideration.

## Conclusion

The data presented herein demonstrated that sodium butyrate manipulates breast cancer cell growth and mediates induction of apoptosis through activation of caspase 3 and 8, enhancement of intracellular ROS level, depletion of mitochondrial membrane potential (Δψm) and induction of cell cycle arrest. Also, our results revealed no significant differences on the effect of sodium butyrate on the aforementioned assays in normal breast cells (MCF10A). Based on our results, no significant differences was observed in responsiveness of estrogen receptor positive (MCF-7) and negative (MDA-MB-468) toward sodium butyrate and apparently further studies are required to clarify the relevance of estrogen receptor in the sodium butyrate-induced apoptosis in breast cancer. Consequently, our data provide insight in to the pro-apoptotic role of sodium butyrate in breast cancer.

## Abbreviations

ANOVA: Non-parametric one-way analysis of variance
DCFH-DA: 2′, 7′-dichlorofluorescin diacetate
DMSO: Dimethylsulfoxide
FBS: Fetal bovine serum
H2O2: Hydrogen peroxide
HAT: Histone acetyltransferases
HDAC: Histone deacetylases
HDACi: Histone deacetylase inhibitors
HEK293: Human embryonic kidney293
MTT: 3-(4,5-dimethyltiazol-2-yl)-2,5-diphenyltetrazolium bromide
NaBu: Sodium butyrate
OH: Hydroxyl radical
PI: Propidium iodide
ROOH: Hydroperoxides
ROS: Reactive oxygen species
TRPM2: Transient receptor potential cation channel, subfamily M, member 2
